# Dysregulation of astrocytic Ca^2+^ signaling and gliotransmitter release in mouse models of α-synucleinopathies

**DOI:** 10.1007/s00401-023-02547-3

**Published:** 2023-02-10

**Authors:** Carmen Nanclares, Jonah Poynter, Hector A. Martell-Martinez, Scott Vermilyea, Alfonso Araque, Paulo Kofuji, Michael K. Lee, Ana Covelo

**Affiliations:** 1 Department of Neuroscience, University of Minnesota, 4-125 Jackson Hall, 321 Church Street SE, Minneapolis, MN 55455 USA; 2 Institute for Translational Neuroscience, University of Minnesota, 2101 6th Street SE, Minneapolis, MN 55455 USA; 3 Institut National de la Santé et de la Recherche Médicale (INSERM), U1215 NeuroCentre Magendie, 33077 Bordeaux, France; 4 University of Bordeaux, 33077 Bordeaux, France

**Keywords:** Astrocyte, Calcium, Gliotransmission, α-synuclein, Synucleinopathies

## Abstract

α-Synuclein is a major component of Lewy bodies (LB) and Lewy neurites (LN) appearing in the postmortem brain of Parkinson's disease (PD) and other α-synucleinopathies. While most studies of α-synucleinopathies have focused on neuronal and synaptic alterations as well as dysfunctions of the astrocytic homeostatic roles, whether the bidirectional astrocyte–neuronal communication is affected in these diseases remains unknown. We have investigated whether the astrocyte Ca^2+^ excitability and the glutamatergic gliotransmission underlying astrocyte–neuronal signaling are altered in several transgenic mouse models related to α-synucleinopathies, i.e., mice expressing high and low levels of the human A53T mutant α-synuclein (G2-3 and H5 mice, respectively) globally or selectively in neurons (iSyn mice), mice expressing human wildtype α-synuclein (I2-2 mice), and mice expressing A30P mutant α-synuclein (O2 mice). Combining astrocytic Ca^2+^ imaging and neuronal electrophysiological recordings in hippocampal slices of these mice, we have found that compared to non-transgenic mice, astrocytes in G2-3 mice at different ages (1–6 months) displayed a Ca^2+^ hyperexcitability that was independent of neurotransmitter receptor activation, suggesting that the expression of α-synuclein mutant A53T altered the intrinsic properties of astrocytes. Similar dysregulation of the astrocyte Ca^2+^ signal was present in H5 mice, but not in I2-2 and O2 mice, indicating α-synuclein mutant-specific effects. Moreover, astrocyte Ca^2+^ hyperexcitability was absent in mice expressing the α-synuclein mutant A53T selectively in neurons, indicating that the effects on astrocytes were cell-autonomous. Consistent with these effects, glutamatergic gliotransmission was enhanced in G2-3 and H5 mice, but was unaffected in I2-2, O2 and iSyn mice. These results indicate a cell-autonomous effect of pathogenic A53T expression in astrocytes that may contribute to the altered neuronal and synaptic function observed in α-synucleinopathies.

**Supplementary Information:**

The online version contains supplementary material available at 10.1007/s00401-023-02547-3.

## Introduction

Parkinson’s disease (PD) and dementia with Lewy Bodies (DLB) are late-onset neurodegenerative diseases characterized by both motor and cognitive symptoms. While the cause of the disease is unknown in most cases, familial PD is caused by abnormalities in selected genes, including the α-synuclein gene (*SNCA*). These abnormalities can be duplication or triplication of the gene [17, 28, 61], but also point mutations, including the A53T [55], A30P [34] and E46K [72]. These and other neurodegenerative dementia in the family of the α-synucleinopathies are characterized by the presence of cytoplasmic inclusions of fibrillary α-synuclein, called Lewy bodies (LB) and Lewy neurites (LN) [64], that are commonly found in neurons but have also been reported in astrocytes [11, 19, 68]. α-synuclein malfunction may contribute to pathogenesis by disrupting cellular homeostasis, synaptic function and finally inducing neuronal death.

Besides housekeeping functions such as buffering of excess potassium and neurotransmitters in the extracellular space, astrocytes have been implicated in the active regulation of neurotransmission at synapses by the release of neuroactive substances termed gliotransmitters [3, 5, 42, 53, 57, 71]. In this view of a tripartite synapse, astrocytes actively respond to neurotransmitters via receptor-induced Ca^2+^ elevations and, in turn, release gliotransmitters (e.g., glutamate) to have feedback actions on neurons and synapses. The investigations on the role of astrocytes in α-synucleinopathies have been centered on their participation in inflammatory responses and it remains unclear whether astrocyte-neuronal communication and synaptic function are also disrupted.

We report here the properties of astrocyte-neuron communication in different mouse models of early-onset familial PD. Using confocal imaging of astrocyte Ca^2+^ activity in combination with electrophysiological recordings from CA1 pyramidal neurons, we found that A53T mutant α-synuclein markedly alters the intrinsic Ca^2+^ activity of astrocytes as well as their gliotransmitter release. Our results also show that these alterations in astrocyte physiological properties are A53T-mutant specific and do not rely on the neuronal expression of α-synuclein. Thus, our results provide novel insights toward understanding on how α-synucleinopathies can lead to aberrant signaling at a tripartite synapse and suggest a novel mechanism for the synaptic dysfunction observed in these diseases.

## Materials and methods

### Ethics statement

All of the procedures for handling and sacrificing animals were approved by the University of Minnesota Institutional Animal Care and Use Committee (IACUC) in compliance with the National Institutes of Health guidelines for the care and use of laboratory animals (#1701A34507).

### Mouse lines

Mice were housed under 12/12-h light/dark cycle and up to five animals per cage. Males and females 1–6-month-old transgenic mice expressing human wild type (WT) α-synuclein (line I2-2), A53T mutant α-synuclein (lines G2-3 and H5), and A30P mutant α-synuclein (line O2) under the control of a mouse prion protein promoter were used in this study [36]. Transgene negative (TgNg) G2-3 littermates were used as control mice. The generation of these mouse lines has been previously described [36]. Mouse genotype was identified by tail DNA genotyping as previously described [36]. Mice from line G2-3 is available from Jackson Laboratories (B6.Cg-*2310039L15Rik*^Tg(Prnp−SNCA*A53T)23Mkle^/J, strain #006,823). In addition, we used mice lacking α-synuclein expression (B6;129X1-*Snca*^*tm1Rosl*^/J, Jax strain #003,692).

Conditional transgenic mice expressing A53T mutant human α-synuclein in forebrain excitatory neurons (iSynAT) were generated by mating CamKIIα-tTA driver mice (B6.Cg-Tg(Camk2a-tTA)1Mmay/DboJ, Jax strain #007004) to Tet-O-hαSyn(A53T) responder mice (Jax: Tg(tetO-SNCA*A53T)E2Cai/J, Jax strain #012443). These iSynAT mice express high levels of transgene expression in forebrain neurons [39].

All mice used were exhaustively backcrossed (at least 10 generations) to C57Bl6/J background and maintained in C57Bl6/J congenic background. The mouse genotype was identified by tail DNA genotyping as previously described [36].

The TgA53T mice (line G2-3) develop subcortical pathology after 10 months of age with very little forebrain pathology [36]. Moreover, these mice do not exhibit any α-synuclein pathology at 6 months of age [60]. In the G2-3 mice, astrocytes in the brainstem and spinal cord are known to harbor α-synuclein pathology along with neuronal pathology [63], but no α-synuclein pathology is seen in cortical or hippocampal astrocytes. In our hands, mice from lines H5, I2-2, and O2 do not develop progressive neurodegenerative disease or overt α-synucleinopathy with aging.

### Hippocampal slice preparation

Animals were decapitated and the brain was rapidly removed and placed in ice-cold artificial cerebrospinal fluid (aCSF). Hippocampal slices (350 μm thick) were made with a vibratome and incubated (> 30 min) at room temperature in aCSF containing (in mM): 124 NaCl, 5 KCl, 1.25 NaH_2_PO_4_, 2 MgSO_4_, 26 NaHCO_3_, 2 CaCl_2_, and 10 glucose, and was gassed with 95% O_2_/5% CO_2_ (pH = 7.3–7.4). Slices were transferred to an immersion recording chamber, superfused at 2 ml/min with gassed aCSF, and visualized under an Olympus FV300 laser-scanning confocal microscope or an Olympus BX50WI microscope (Olympus Optical, Japan).

### Electrophysiology

Electrophysiological recordings from CA1 pyramidal neurons were made in whole-cell configuration of the patch-clamp technique. Patch electrodes had resistances of 5–10 MΩ when filled with the internal solution containing (in mM): 117 cesium-gluconate, 20 HEPES, 0.4 EGTA, 2.8 NaCl, 5 TEA-Cl, 2 ATP-Mg^2+^, and 0.3 GTP-Na^+^ (pH = 7.3). Recordings were obtained with a PC-ONE amplifier (Dagan Instruments, MN, US) using a DigiData 1440A. Signals were filtered at 1 kHz and acquired at a 10 kHz sampling rate. Membrane potential was held at − 70 mV. The pCLAMP 10.4 (Axon Instruments) software was used for data display, acquisition, and storage. Miniature excitatory postsynaptic currents (mEPSCs) and slow inward currents (SICs) were isolated in the presence of tetrodotoxin (TTX, 1 µM), picrotoxin (50 µM), and CGP54626 (1 µM) to block voltage-gated Na^+^ channels, GABA_A,_ and GABA_B_ receptors, respectively. Miniature inhibitory postsynaptic currents (mIPSCs) were isolated in the presence of tetrodotoxin (TTX, 1 µM), CNQX (20 μM) and AP5 (50 μM) to block voltage-gated Na^+^ channels, AMPA receptors and NMDA receptors, respectively. Recordings of mIPSCs were performed at a holding voltage of 0 mV.

### Ca^2+^ imaging

Ca^2+^ levels in astrocytes located in the *stratum radiatum* of the CA1 region of the hippocampus were monitored using the Ca^2+^ indicator fluo-4. Astrocytes were loaded with the dye by incubating the slices with fluo-4-AM (2 μM and 0.01% of pluronic) for 45–60 min at room temperature [4, 31, 47, 49, 50]. When fluo-4-positive cells were patched and characterized by their electrophysiological properties they presented typical astrocytes characteristics (*n = *9), this is, low input resistance, a quasi-linear voltage–current relationship under voltage-clamped conditions and absence of action potentials (Supplementary Fig. 1a-c). On the contrary, recordings from fluo-4-negative cells in the *stratum radiatum* exhibited typical neuronal characteristics (*n = *14) such as higher input resistances, a non-linear voltage–current curve and fired action potentials (Supplementary Fig. 1d-f). Additionally, slices incubated with fluo-4 and sulforhodamine 101 (SR101), a widely used astrocyte marker known to label astrocytes in the hippocampus [58], provided good co-localization between SR101 and fluo-4-positive cells (Supplementary Fig. 1g). Thus, we conclude that, with the protocols used in the present study, we analyzed the Ca^2+^ activity of hippocampal astrocytes. Recordings were done in the presence of TTX 1 µM (unless otherwise indicated) in order to minimize the contribution of neuronal activity to the astrocyte Ca^2+^ activity. Astrocytes were imaged using epifluorescence (Olympus BX50WI microscope) or confocal microscopy (Olympus FV300 laser-scanning confocal microscope; image acquisition rate: 1 Hz). ImageJ software (NIH) was used for quantitative epifluorescence measurements. Ca^2+^ variations recorded at the soma of the cells were estimated as changes of the fluorescence signal over baseline (ΔF/F_0_), and cells were considered to show a Ca^2+^ event when ΔF/F_0_ increased two times the standard deviation of the baseline. The astrocyte Ca^2+^ signal was quantified from the astrocyte Ca^2+^ event frequency, which was calculated from the number of Ca^2+^ events per min within 10 min of recording. Astrocyte Ca^2+^ events were obtained from 5 to 25 astrocytes in the field of view during the recording.

### Primary astrocyte cultures

Cerebral cortices from postnatal day 0–2 mouse pups were dissected out and placed in cold Hibernate-A medium (BrainBits LLC). Cortices were transferred to a digestion medium containing Hibernate-A -CaCl_2_ (BrainBits LCC), Papain, l-Cysteine, and EDTA for 12 min at 37ºC followed by a wash in a digestion inhibitor solution and gentle trituration. The cells were plated in a growing medium containing DMEM, Sodium Pyruvate, GlutaMAx, Penicillin–Streptomycin and Fetal Bovine Serum at a density of 3 million cells per T25 flask. Mixed glia cultures were left growing for at least 10 days. Cultures were then shaken at 200 rpm for 24 h at 37ºC. The culture media was then replaced with PBS followed by a 7 min trypsin incubation at 37ºC to lift the astrocyte monolayer. For protein analysis, astrocytes were plated in NbAstro (BrainBits LCC), at a density of 100 k cells per well of a Poly-D-Lysine-coated 12-well plate.

### Gel electrophoresis and immunoblotting

Primary cortical astrocytes were lysed using ice-cold complete TNE (50 mM Tris–HCl, 150 mM NaCl, 5 mM EDTA, 1% sodium dodecyl sulfate, 0.5% Nonidet P-40, 0.5% sodium deoxycholate) and HALT protease and phosphatase inhibitors (Thermo-Fisher; Waltham, MA). Cell homogenates were sonicated, boiled and centrifuged for 10 min at 16,000 xg. Protein concentration was estimated using BSA assay (Thermo Fisher). Concentration corrected protein samples were diluted in reducing sample buffer (Boston BioProducts), run on 4–20% Criterion TGX gels (Bio-Rad) and transferred onto 0.45 µm nitrocellulose membranes. Protein on membranes were probed using the following primary antibodies: total α-synuclein (BD Biosciences 610,787), human α-synuclein (HuSyn1) (Made by Lee MK et al., 2002), and α-tubulin (Abcam ab4074). Membranes were visualized using chemiluminescent substrates (Thermo Scientific) and the ImageQuant LAS 4000 detection system (GE Healthcare).

### Immunohistochemical staining

Brain tissues were isolated from G2-3 end stage mice as well as TgNg controls and post-fixated in 4% paraformaldehyde for further processing and paraffin embedding. Sections were cut at a thickness of 7 µm. Immunostaining was performed using the immunoperoxidase method with diaminobenzidine. After deparaffinization followed by antigen retrieval using Reveal Decloaker (Biocare Medical), sections were blocked for 10 min using Background Sniper (Biocare Medical), followed by incubation in primary antibodies (Human-specific α-synuclein, see [36]; GFAP, Synaptic Systems #173–006; GFAP, DakoCytomation Z0334; phospho aS, WAKO, #015–25,191) at 4ºC overnight. Sections were then incubated in fluorescent conjugated secondary antibodies for 2 h. Images were captured using a Leica Microsystems Stellaris confocal system.

### Statistics

Data are expressed as mean ± standard error of the mean (s.e.m.). Data normality was assessed using a Kolmogorov–Smirnov statistical test. When data met a normal distribution results were compared using a two-tailed Student’s *t*-test (α = 0.05), One-way ANOVA, or Two-way ANOVA with Tukey method post hoc, otherwise, data were compared using a Mann–Whitney test or Kruskal–Wallis One-Way ANOVA with Dunn’s method post hoc. Statistical differences were established with *p < *0.05 (*), *p < *0.01 (**) and *p < *0.001 (***). For the cumulative probability data, results were compared using a two-sample Kolmogorov–Smirnov statistical test using the following equation

$${D}_{\alpha }=c(\alpha )\sqrt{\frac{{n}_{1}+ {n}_{2}}{{n}_{1}{n}_{2}},}$$

where *D*_*α*_ is the critical *D* value (maximum difference between the cumulative distributions), *n*_1_ and *n*_2_ are the two samples of sizes and the coefficient is given by the table below.

**Table:** 

*α*	0.10	0.05	0.025	0.01	0.005	0.001
*c* (*α*)	1.22	1.36	1.48	1.63	1.73	1.95

### Drugs and chemicals

Flupenthixol dihydrochloride, CGP54626 hydrochloride, suramin hexasodium salt, AM 251, D-AP5, CNQX disodium salt, LY367385, MPEP hydrochloride, and Tetrodotoxin (TTX) were purchased from Tocris Bioscience. Picrotoxin was purchased from Indofine Chemical Company (Hillsborough, NJ). Fluo-4-AM from Molecular Probes (Eugene, OR). All other drugs were purchased from Sigma.

## Results

### Astrocyte Ca^2+^ signal is altered in G2-3 transgenic mice

**Fig. 1:**
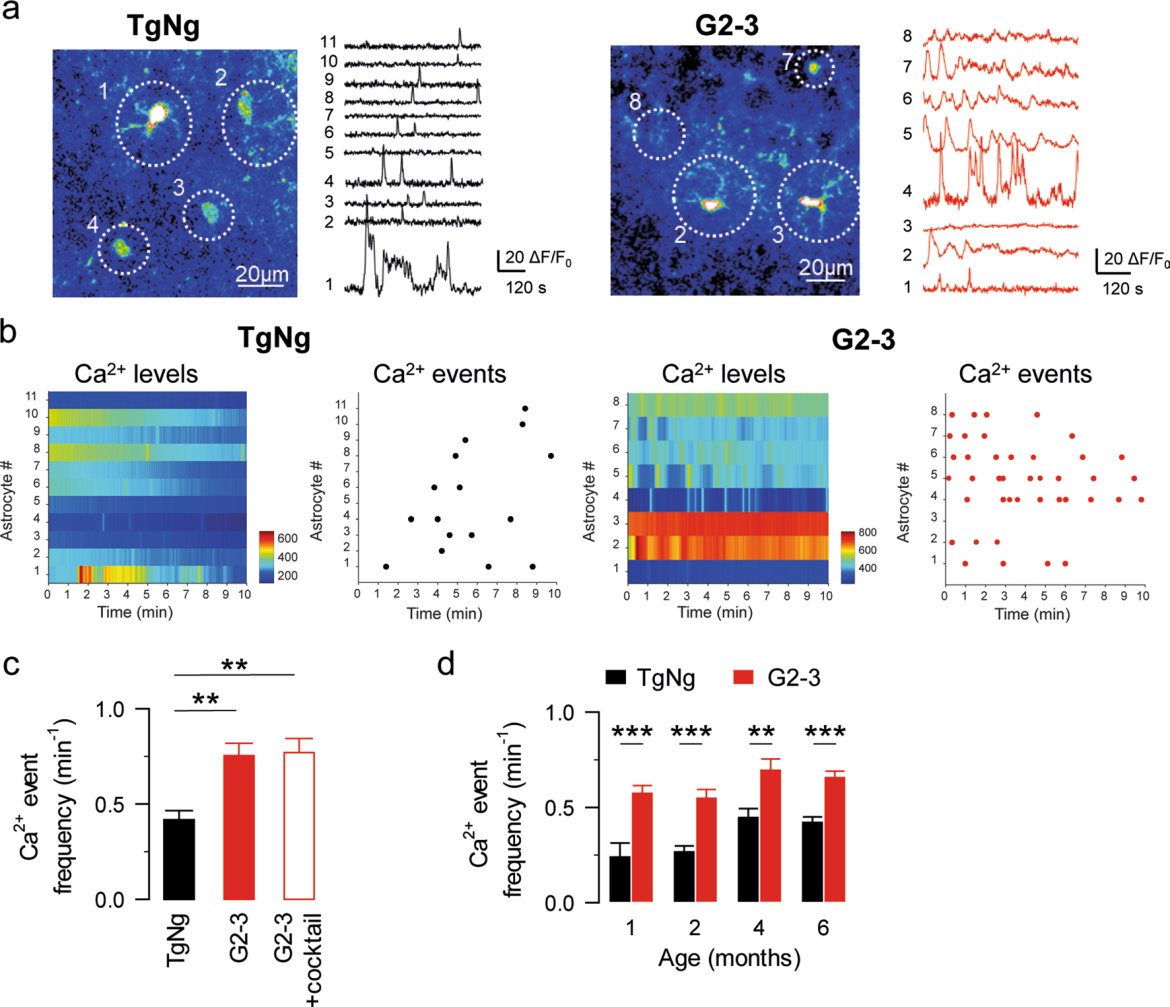
Astrocyte Ca^2+^ signal is altered in G2-3 transgenic mice. **a** Pseudocolor images and representative traces of Ca^2+^ fluorescence intensities obtained from transgene negative (TgNg) and G2-3 mice. **b** Heat map indicating Ca^2+^ levels and raster plot indicating Ca^2+^ events along time obtained from TgNg and G2-3 mice. **c** Ca^2+^ events per minute obtained from TgNg and G2-3 mice in control conditions and in the presence of a cocktail of antagonists. **d** Ca^2+^ events per minute obtained from TgNg and G2-3 mice at different ages. Data are expressed as mean ± s.e.m. (**)*p < *0.01, (***)*p < *0.001

Astrocyte excitability is encoded by fluctuations of cytosolic Ca^2+^ variations that occur spontaneously or in response to neurotransmitters released during neuronal activity [3, 33]. The A53T mutation in the *SNCA* gene is one common cause of early-onset familial PD [55]. To investigate the effects of human A53T-mutant α-synuclein on the spontaneous astrocyte Ca^2+^ activity, we examined the astrocyte Ca^2+^ signals from hippocampal astrocytes in brain slices from 5–6-month-old G2-3 mice (a transgenic mouse line that expresses human α-synuclein with the A53T point mutation, Table 1) [36] and from their transgene negative littermates (TgNg). Hippocampal slices were loaded with the Ca^2+^ indicator Fluo-4, and astrocytes in the CA1 region were imaged for 10 min. Because astrocytes can respond to neurotransmitters released by neurons [3, 33, 71], to minimize neuronal contribution to the astrocyte Ca^2+^ activity, experiments were performed in the presence of tetrodotoxin (TTX 1 µM) which inhibits the firing of action potentials by blocking voltage-gated sodium channels. We found that the astrocyte Ca^2+^ event frequency (calculated as the number of Ca^2+^ events per minute) was significantly higher in astrocytes from the G2-3 transgenic mice (0.76 ± 0.07 events per minute, *n = *93 astrocytes from *n = *10 slices; Fig. 1a-c) than those from the TgNg mice (0.42 ± 0.05 events per minute, *n = *68 astrocytes from *n = *9 slices, *p = *0.007; Fig. 1a-c). Interestingly, these changes in astrocyte Ca^2+^ event frequency were present in animals as young as 1 month of age (Fig. 1d), indicating that the astrocyte signal is altered in G2-3 mice early in their lifespan.

**Table 1:** Summary of mouse lines

Mouse line	Type of α-synuclein
TgNg	Transgene negative
I2-2	Human wild type α-synuclein
G2-3	High expression levels of human A53T mutant α-synuclein
H5	Low expression levels of human A53T mutant α-synuclein
O2	Human A30P mutant α-synuclein
iSyn	Neuronal expression of human A53T mutant α-synuclein
SynKO	α-synuclein knockout

Mouse lines used in this study and their expression of α-synuclein

**Fig. 2:**
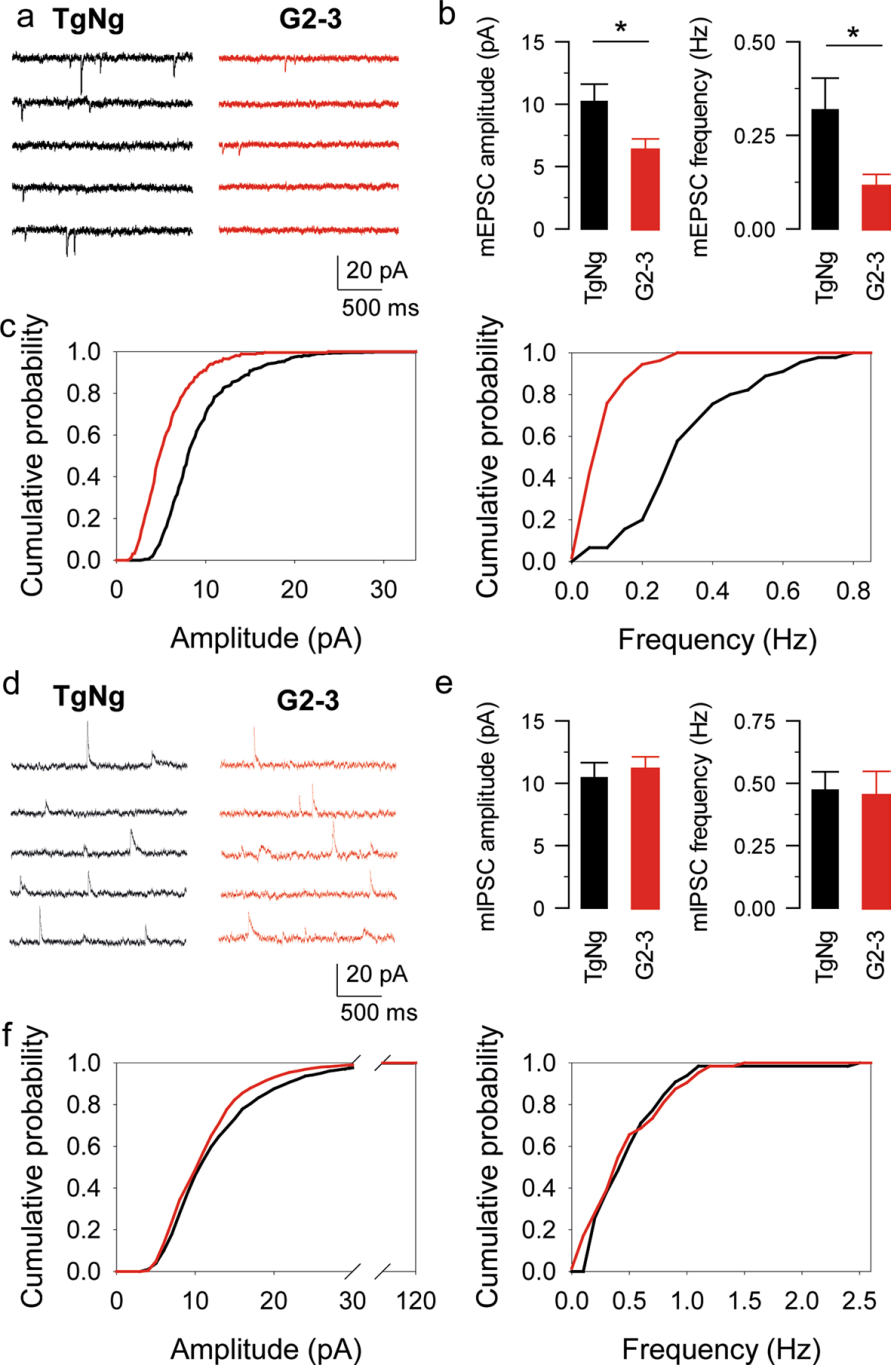
The increase in astrocyte Ca^2+^ event frequency observed in the G2-3 mice is not a consequence of an increase in neurotransmitter release. **a** mEPSC traces recorded from TgNg and G2-3 mice. **b** mEPSC amplitude and frequency obtained from TgNg and G2-3 mice. Data are expressed as mean ± s.e.m. (*) *p < *0.05. **c** Cumulative probability of mEPSC amplitude (left; the maximum difference between the cumulative distributions, D, is 0.44781 with a corresponding *p < *0.001) and mEPSC frequency (right; the maximum difference between the cumulative distributions, D, is 0.74444 with a corresponding *p < *0.001). **d** mIPSC traces recorded from TgNg and G2-3 mice. **e** mIPSC amplitude and frequency obtained from TgNg and G2-3 mice. Data are expressed as mean ± s.e.m. **f** Cumulative probability of mIPSC amplitude (left; the maximum difference between the cumulative distributions, D, is 0.09153 with a corresponding *p > *0.1) and mIPSC frequency (right; the maximum difference between the cumulative distributions, D, is 0.17188 with a corresponding *p > *0.1)

Astrocytes express a variety of receptors that allow them to respond to neurotransmitters with Ca^2+^ increases [2, 3, 21, 22, 27, 41, 45, 48, 52, 53, 70, 71]. We next investigated the possibility that the increase in astrocyte Ca^2+^ event frequency observed in the G2-3 mice is a readout of an increase in neurotransmitter release. To test this possibility, we first monitored astrocyte Ca^2+^ activity in the presence of a cocktail of neurotransmitter receptor antagonists containing CNQX 20 μM, AP5 50 μM, MPEP 50 μM, LY367385 100 μM, picrotoxin 50 μM, CGP54626 1 μM, atropine 50 μM, CPT 10 μM, suramin 100 μM, flupenthixol 30 μM and TTX 1 μM to block glutamatergic, GABAergic, cholinergic, purinergic and dopaminergic transmission. Under these conditions, astrocyte Ca^2+^ event frequency was still increased in G2-3 mice (0.77 ± 0.07 events per minute, *n = *87 astrocytes from *n = *8 slices, *p = *0.007; Fig. 1c) indicating that the increase in astrocyte Ca^2+^ activity is not due to changes in synaptic signaling. This result is further supported by the observation that both the amplitude and frequency of mEPSCs recorded from CA1 pyramidal neurons are decreased in G2-3 mice compared to their TgNg littermates (amplitude: 10.2 ± 1.4 and 6.32 ± 0.9 pA in TgNg and G2-3 mice, respectively, *p = *0.033. Frequency: 0.32 ± 0.09 and 0.11 ± 0.03 Hz in TgNg and G2-3 mice, respectively, *p = *0.013; *n = *9 and *n = *9 in TgNg and G2-3 mice; Fig. 2a–c) [69], indicating that an increase in the excitatory synaptic activity cannot account for the increase in the astrocyte Ca^2+^ event frequency. Finally, we studied the possibility of an alteration in the inhibitory synaptic transmission. We observed no change in neither the amplitude nor the frequency of mIPSCs recorded from CA1 pyramidal neurons of G2-3 mice and TgNg littermates (amplitude: 10.44 ± 1.22 and 11.27 ± 0.97 pA in TgNg and G2-3 mice, respectively, *p = *0.505. Frequency: 0.47 ± 0.07 and 0.45 ± 0.10 Hz in TgNg and G2-3, respectively, *p = *0.856; *n = *13 and *n = *13 in TgNg and G2-3 mice; Fig. 2d–f). These data indicate that the observed increase in astrocyte Ca^2+^ activity in G2-3 mice is not due to an increase in neurotransmission.

### Astrocyte Ca^2+^ signal alterations are A53T mutant-specific

**Fig. 3:**
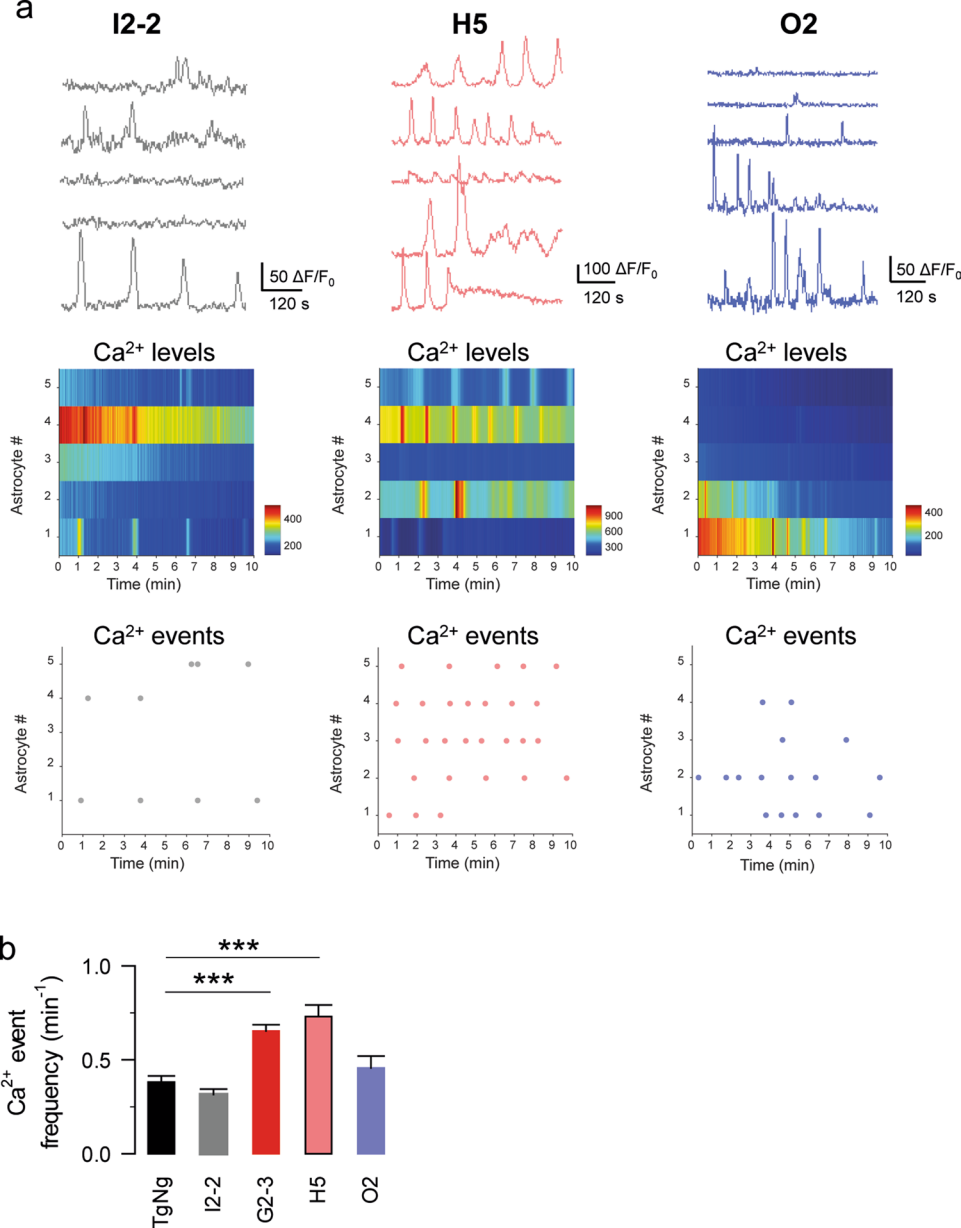
Astrocyte Ca^2+^ alterations are A53T-mutant specific. **a** Representative Ca^2+^ traces, heat maps and raster plots obtained from astrocytes in I2-2, H5 and O2 mice. **b** Ca^2+^ events per minute obtained from TgNg, I2-2, G2-3, H5 and O2 mice. Data are expressed as mean ± s.e.m. (***) *p < *0.001

In neurons, increased expression of α-synuclein per se cause presynaptic inhibition of neurotransmitter release while only A53T mutant α-synuclein causes postsynaptic deficits [69]. Thus, we next investigated whether the observed differences in astrocyte Ca^2+^ event frequency were specific for the A53T mutant α-synuclein or to the overexpression of human α-synuclein in general. To do this, we first used the I2-2 mouse line that expresses a human WT form of α-synuclein (Table 1) [36, 69]. We found that the astrocyte Ca^2+^ event frequency in slices obtained from I2-2 mice was not significantly different from that of TgNg mice (0.32 ± 0.03 events per minute, *n = *65 astrocytes from *n = *10 slices; Fig. 3), indicating that the observed changes in astrocyte Ca^2+^ event frequency in G2-3 mice were probably due to the A53T mutation. To further test this hypothesis, we used a transgenic mouse line (H5) that expresses lower levels of human A53T-mutant α-synuclein than the G2-3 mouse line (Table 1) [36, 69]. We observed that the astrocyte Ca^2+^ event frequency was increased when compared with TgNg mice (0.73 ± 0.06 events per minute, *n = *89 astrocytes from *n = *10 slices, *p < *0.001; Fig. 3) and was similar to that observed in G2-3 mice (Fig. 3). Altogether, these data suggest that the observed increase in astrocytic Ca^2+^ event frequency in the G2-3 transgenic mice was not due to the overexpression of human α-synuclein, but was probably due to the presence of the A53T mutation.

We next explored whether other types of mutant α-synucleins also related to familial PD induce similar changes in the astrocytic Ca^2+^ event frequency. To do this, we analyzed the astrocyte Ca^2+^ signal in the O2 transgenic mice, which expresses human α-synuclein with the A30P mutation (Table 1). We previously showed that despite very high levels of expression, A30P mutant do not cause postsynaptic deficits in neurons [69]. The astrocytic Ca^2+^ event frequency in these mice was not significantly different than that of the TgNg mice (0.46 ± 0.06 events per minute, *n = *49 astrocytes from *n = *10 slices, *p = *0.842; Fig. 3), suggesting that the observed increase in astrocytic Ca^2+^ event frequency in the transgenic mice from G2-3 and H5 lines was A53T mutation-specific.

### Astrocyte glutamate release is increased in G2-3 transgenic mice

**Fig. 4:**
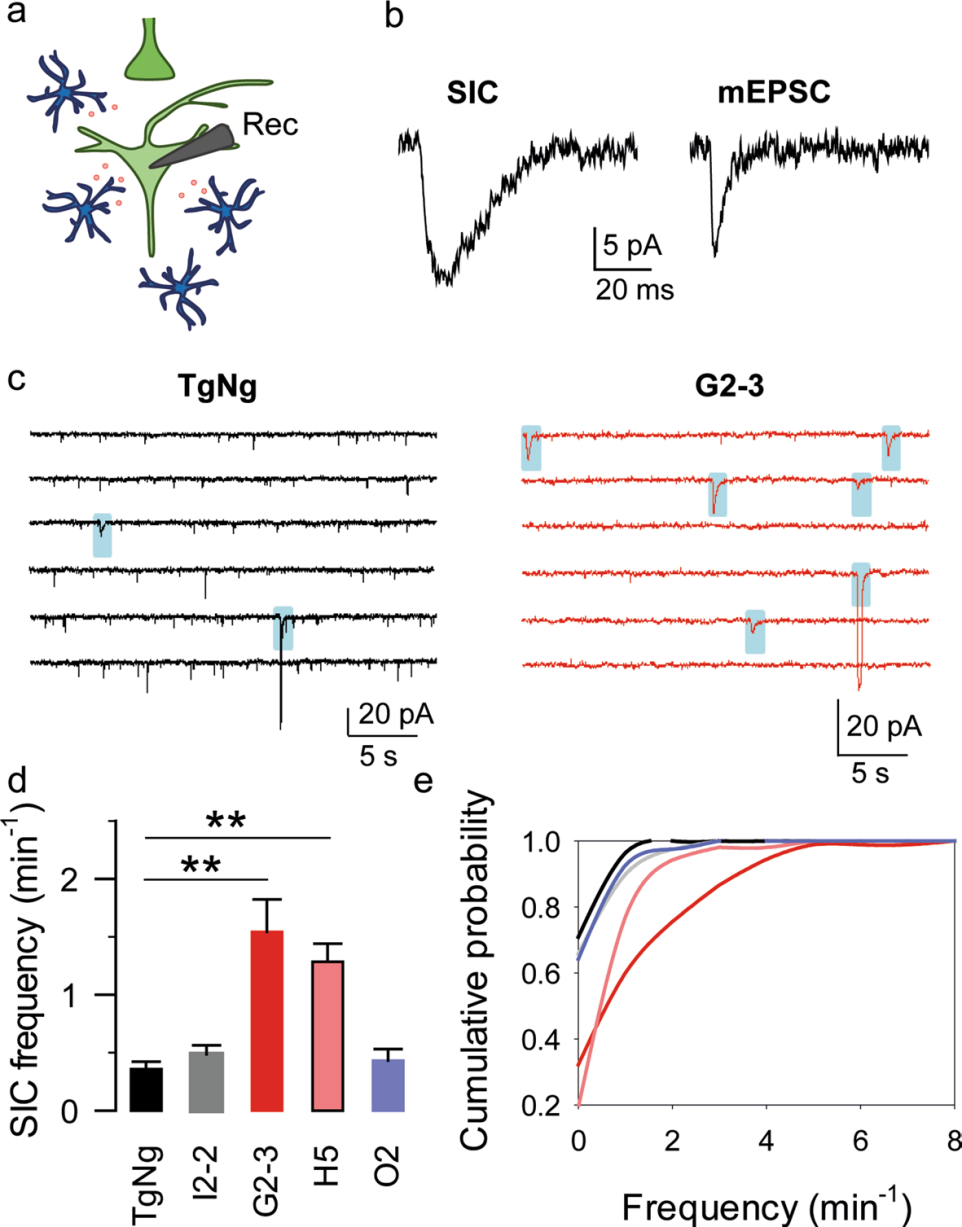
Astrocytic glutamate release is increased in G2-3 mice. **a** Scheme of the experimental approach. Note the glutamate (red) release by astrocytes (blue) and the recording CA1 pyramidal neuron (light green). **b** Representative SIC and mEPSC traces. **c** Representative SIC traces (shaded in blue) obtained from TgNg and G2-3 mice. **d** SICs per minute obtained from TgNg, I2-2, G2-3, H5 and O2 mice. Data are expressed as mean ± s.e.m. (**) *p < *0.01. **e** Cumulative SIC frequency obtained from TgNg, I2-2 (the maximum difference between the cumulative distributions from TgNg and I2-2 mice, D, is 0.064902 with a corresponding *p > *0.1), G2-3 (the maximum difference between the cumulative distributions from TgNg and G2-3 mice, D, is 0.386869 with a corresponding *p > *0.1), H5 (the maximum difference between the cumulative distributions from TgNg and H5 mice, D, is 0.516783 with a corresponding *p > *0.05) and O2 (the maximum difference between the cumulative distributions from TgNg and O2 mice, D, is 0.067116 with a corresponding *p > *0.1) mice

Astrocytes release neuroactive substances, called gliotransmitters [71], which regulate synaptic transmission and neuronal excitability [3, 18, 21, 26, 49, 53]. We next examined whether gliotransmitter release is also altered in G2-3 transgenic mice. To do this, we performed electrophysiological recordings of CA1 pyramidal neurons from G2-3 transgenic and TgNg littermates, and monitored the presence of the NMDA receptor-mediated slow inward currents (SICs), as a biological assay for astrocytic glutamate release [3, 22, 23, 25, 44, 59] (Fig. 4a). SICs were distinguished from mEPSCs by their significantly slower kinetics as previously described [23, 51] (Fig. 4b). We found an increase in SICs frequency in G2-3 transgenic mice (1.54 ± 0.28 SICs per minute, *n = *9 neurons) compared to TgNg mice (0.36 ± 0.06 SICs per minute, *n = *7 neurons, *p = *0.007; Fig. 4c–e), with no changes in SICs duration (195.68 ± 37.17 ms and 194.07 ± 63.86 ms in G2-3 and TgNg mice, respectively; *p = *0.302) or amplitude (7.56 ± 1.34 pA and 9.53 ± 2.36 pA in G2-3 and TgNg mice, respectively; *p = *0.992). Similarly, we found that SICs frequency was also increased in slices obtained from H5 mice, which expresses lower levels of human A53T-mutant α-synuclein [36] (1.29 ± 0.16 SICs per minute, *n = *9 neurons, *p = *0.008; Fig. 4d, e). On the contrary, we did not find changes in SICs frequency in the I2-2 mice (0.49 ± 0.08 SICs per minute, *n = *9 neurons; Fig. 4d, e), that overexpress human WT α-synuclein, or in O2 mice (0.43 ± 0.1 SICs per minute, *n = *10 neurons; Fig. 4d, e), which express human α-synuclein with the A30P mutation. Altogether, these data indicate that glutamate release from astrocytes is increased in an A53T-mutant specific manner. These results are in line with the observation that Ca^2+^ event frequency is also increased in G2-3 and H5 mice, but not in I2-2 and O2 mice (see Fig. 3).

### Alterations in astrocyte–neuron communication are not mediated by neuronal α-synuclein

**Fig. 5:**
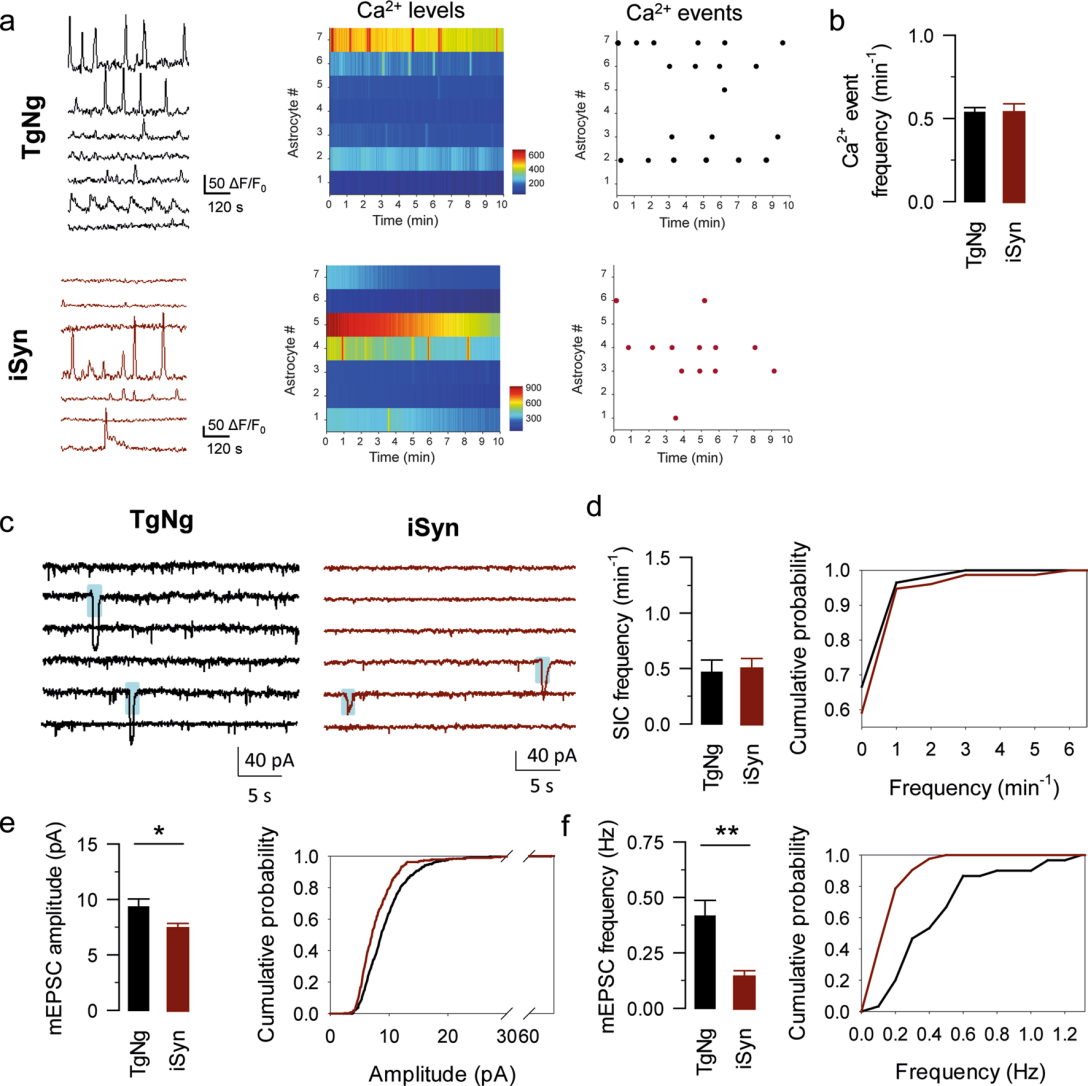
Astrocyte–neuron signaling is not altered by neuronal α-synuclein. **a** Representative Ca^2+^ fluorescence traces obtained from TgNg and iSyn mice (left), heat maps indicating Ca^2+^ levels from TgNg and iSyn mice (middle), raster plots indicating Ca^2+^ events from TgNg and iSyn mice (right). **b** Ca^2+^ events per minute obtained from TgNg and iSyn mice. **c** Representative SIC (shaded in blue) and mEPSC traces obtained from TgNg and iSyn mice. **d** SICs per minute obtained from TgNg and iSyn mice (left), cumulative SIC frequency obtained from TgNg and iSyn mice (right; the maximum difference between the cumulative distributions, D, is 0.074561 with a corresponding *p > *0.1). **e** mEPSC amplitude (left) and cumulative amplitude (right; the maximum difference between the cumulative distributions, D, is 0.22012 with a corresponding *p < *0.001) obtained from TgNg and iSyn mice. **f** mEPSC frequency (left) and cumulative frequency (right; the maximum difference between the cumulative distributions, D, is 0.58571 with a corresponding *p < *0.025) obtained from TgNg and iSyn mice. Data are expressed as mean ± s.e.m. (*) *p < *0.05 and (**) *p < *0.01

Predominant expression of transgenic α-synuclein in neurons of G2-3 model would suggest that neuronal α-synuclein may impact astrocytes, potentially by releasing α-synuclein oligomers that impact astrocyte function [40, 56]. However, expression of mutant α-synuclein in astrocytes from G2-3 model raises the possibility that abnormal α-synuclein in astrocytes causes dysregulation of astrocyte-to-neuronal signaling. To test this hypothesis, we next investigated whether astrocyte–neuron signaling is altered in the transgenic mouse line that specifically overexpresses human A53T-mutant α-synuclein in forebrain excitatory neurons (iSyn mice, Table 1). We recorded astrocyte Ca^2+^ signals and SICs in slices obtained from iSyn mice and we found no difference in astrocytic Ca^2+^ event frequency between the iSyn transgenic mice (0.54 ± 0.053 events per minute, *n = *105 astrocytes from *n = *17 slices) and their TgNg littermates (0.53 ± 0.03 events per minute, *n = *148 astrocytes from *n = *16 slices, *p = *0.24; Fig. 5a, b). In addition, we did not find a difference in SICs frequency in iSyn mice (0.5 ± 0.09 SICs per minute, *n = *9) when compared to their TgNg littermates (0.46 ± 0.11 SICs per minute, *n = *8, *p = *0.832; Fig. 5c, d). Interestingly, mEPSC amplitude and frequency were still reduced in iSyn mice (Amplitude: 9.33 ± 0.72 and 7.38 ± 0.47 pA in TgNg and iSyn mice, respectively, *p = *0.036. Frequency: 0.42 ± 0.07 and 0.14 ± 0.03 Hz in TgNg and iSyn mice, respectively, *p = *0.002; *n = *8 and *n = *9 in TgNg and iSyn mice, respectively; Fig. 5c, e, f). These data suggest that, although neuronal deficits are still present, the enhanced astrocyte-neuron signaling observed in the G2-3 mice is not mediated by the transfer of α-synuclein aggregates from neurons to astrocytes.

**Fig. 6:**
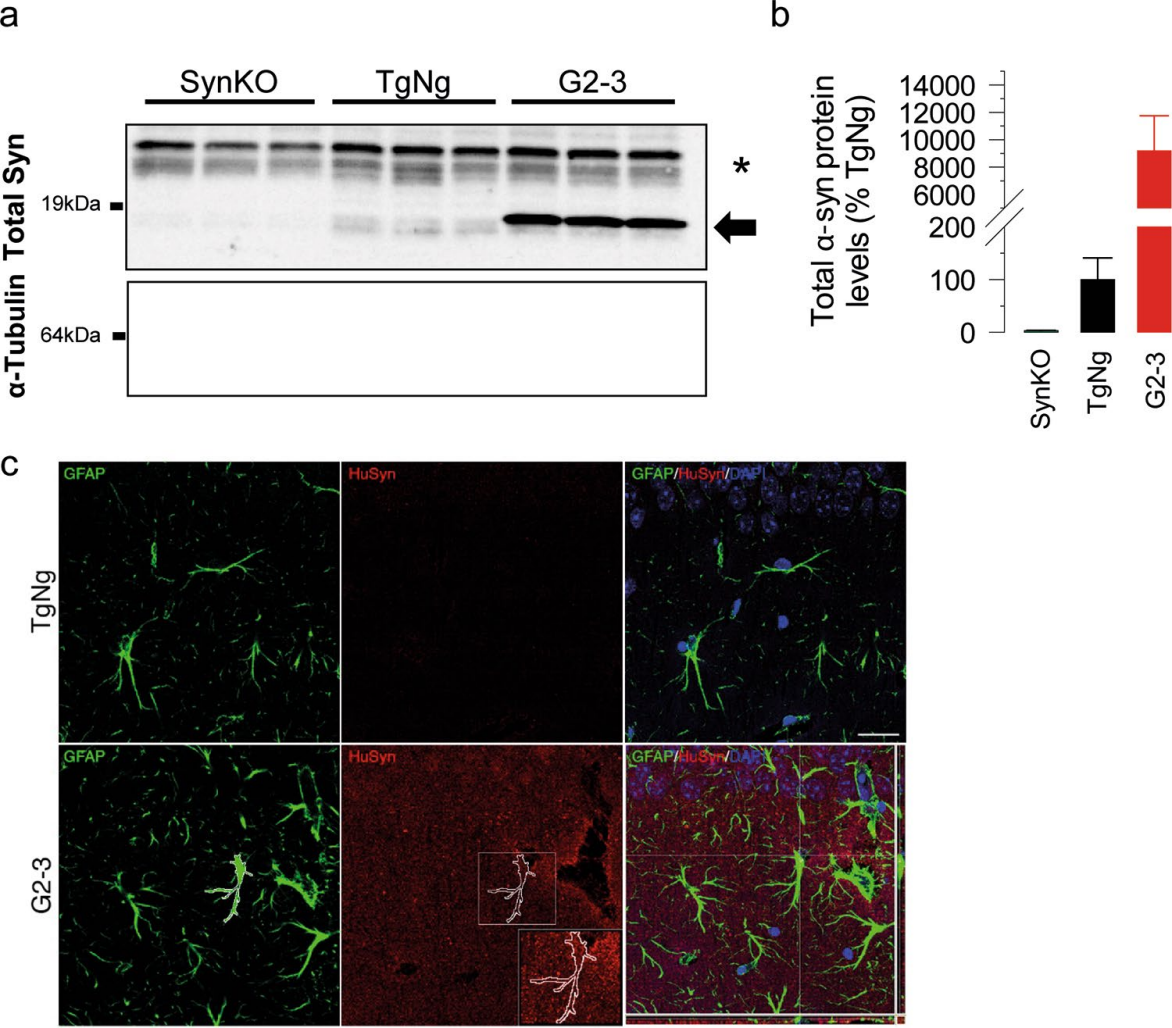
Analysis of α-synuclein expression in astrocytes. Lysates from primary astrocyte cultures from brains of SynKO, TgNg and G2-3 mice were analyzed for α-synuclein expression. **a** Immunoblot for α-synuclein from brain samples from SynKO, TgNg and G2-3 mice. α-tubulin is used as a loading control. Note that G2-3 astrocytes show strong α-syn reactivity (arrow). α-syn is present in TgNg astrocytes but not in SynKO astrocytes. *Non-specific band. **b** α-synuclein protein levels quantified from a. Data was normalized to α-tubulin levels and relativized to TgNg. **c** Astrocytes within the *stratum radiatum* of G2-3 mice, but not TgNg mice, contain intracellular human α-syntransgenic negative; *TgNg* glial fibrillary acidic protein, *GFAP* human alpha-synuclein, HuSyn. Scale bar = 25 μm

Collectively, our results suggest that neuronal expression of the A53T mutant α-synuclein is not sufficient to alter astrocyte–neuron communication. Thus, we tested if the transgene in the G2-3 model is expressed in astrocytes. Specifically, while transgene in the G2-3 model is highly expressed in neurons, the promoter used (moPrp) can drive expression in non-neural tissues [9] and mouse Prp is expressed in glial cells [43]. To determine if A53T mutant human α-synuclein is expressed in astrocytes, we established primary cultures of astrocytes from G2-3 and TgNg mice, along with α-synuclein knockout mice (SynKO), and performed immunoblot analysis for α-synuclein expression. We show that α-synuclein is expressed in astrocytes from TgNg and G2-3, but not SynKO mice (Fig. 6a, b). Using human α-synuclein specific antibody, we also verified that human α-synuclein expression is expressed by G2-3 astrocytes, albeit at much lower levels than in neurons (data not shown). We also demonstrate the presence of human α-synuclein inside G2-3 astrocytes, but not TgNg hippocampal astrocytes in the *stratum radiatum* (Fig. 6c) and the *dentate gyrus* (Supplementary Fig. 2). Based on these results, we propose that abnormal α-synuclein in astrocytes causes the dysregulation of astrocyte-to-neuronal signaling.

## Discussion

α-synucleinopathies are characterized by abnormalities in α-synuclein that lead to synaptic dysfunction, neuronal loss and motor and cognitive deficits. While the effects of α-synuclein on neurons have been studied extensively, its effect on astrocyte physiology remains largely unknown. Here, we investigated the impact of human A53T-mutant α-synuclein on astrocyte-neuron communication. Our results showed that both astrocyte Ca^2+^ activity and gliotransmission were enhanced in mice expressing human A53T-mutant α-synuclein but not in mice expressing human WT or A30P-mutant α-synuclein, indicating that specifically, the A53T point mutation exacerbates astrocyte-neuron signaling. Interestingly, these alterations in astrocyte–neuron communication were independent of neurotransmission and were not present in mice expressing human A53T-mutant α-synuclein exclusively in neurons, suggesting a cell-autonomous effect of A53T-mutant α-synuclein in astrocyte Ca^2+^ signaling and gliotransmitter release.

Unlike neurons, astrocytes are non-electrically excitable cells; instead, astrocyte excitability is encoded by Ca^2+^ fluctuations that occur spontaneously but also in response to neighboring neuronal activity [3, 21, 48]. Our results show that astrocyte Ca^2+^ activity from mice that express human A53T-mutant α-synuclein is altered independently of neurotransmission indicating that A53T mutant α-synuclein directly impacts the intrinsic Ca^2+^ activity of astrocytes. Previous evidence has shown that α-synuclein aggregation produces long-lasting Ca^2+^ transients in cultured neurons [7, 15, 16, 38] and astrocytes [1]. Although both WT and mutant forms of α-synuclein can self-aggregate at high concentrations, the A53T mutant form can aggregate with faster kinetics [6, 37], suggesting that the observed Ca^2+^ alterations may be due to the efficient formation of A53T-mutant α-synuclein aggregations. Even though the specific mechanism remains to be elucidated, our results demonstrate that the A53T-mutant α-synuclein alters the astrocyte Ca^2+^ signal in situ, adding up to other studies showing dysfunctional astrocyte Ca^2+^ signaling in various neurological disorders, including other models of PD [10], Alzheimer’s disease (AD) [20, 24, 35, 65] and Huntington’s disease [29].

Astrocytes regulate synaptic transmission and neuronal excitability by releasing gliotransmitters [3]. Glutamate released from astrocytes enhances postsynaptic excitability and neuronal coordination by activating extrasynaptic NMDA receptors and inducing SICs [3, 22, 23, 25, 44, 59]. Abnormal release of glutamate and GABA from astrocytes has been observed in models of AD contributing to enhanced neuronal excitability, synapse loss, synaptic plasticity deficits, and memory impairment [24, 30, 54, 66]. Our results show that SICs frequency was enhanced in mice expressing human A53T-mutant α-synuclein, indicating that astrocyte-neuron signaling is also increased in α-synucleinopathies. No changes in SIC frequency were found in mice expressing human WT or A30P-mutant α-synuclein, suggesting that the increased gliotransmission may be also linked to the high aggregation potential of the A53T-mutant form of α-synuclein [6].

α-synuclein is a cytosolic protein that can associate with the plasma membrane and is enriched in presynaptic terminals. Through its interaction with the SNARE complex and other presynaptic proteins [12–14], α-synuclein supports synaptic function and suppresses vesicle release by depleting the recycling and readily releasable pools of synaptic vesicles [46]. In addition, human A53T-mutant α-synuclein also causes postsynaptic and memory deficits by its interaction with the microtubule-associated protein tau [60, 69]. The results reported here show that, in addition to the pre- and postsynaptic deficits, A53T-mutant α-synuclein promotes chronic glutamate release from astrocytes. The hyperfunction of extrasynaptic NMDARs promoted by astrocytic glutamate release may cause neuronal excitotoxicity as postulated to many neurodegenerative diseases. In addition, the dysregulated gliotransmitter release may influence the spatiotemporal dynamics of neuronal networks and thus contributing to the pathology observed in α-synucleinopathies.

While α-synuclein is abundantly expressed in neurons, studies show that astrocytes express low levels of SNCA [8, 32, 62, 67, 73]. In addition to evidence of astrocyte expression of α-synuclein [62, 67], α-synuclein-positive inclusions have been found in astrocytes in postmortem PD brains [8]. Here, we show that astrocytes from G2-3 mice express much higher levels of α-synuclein than normal (see Fig. 6). While the very high levels of human α-synuclein expression could contribute to the abnormal astrocyte neurotransmission, increased expression alone is not likely to be sufficient. Specifically, expression of lower levels of transgene in line H5 [69] leads to astrocytes abnormalities (see Figs. 3, 4), while the high level expression of wild type (line I2-2) or the A30P mutant (line O2) human α-synuclein did not impact astrocyte neurotransmission (Figs. 3, 4). In addition, increased expression of α-synuclein by astrocytes may also be a feature of PD as astrocytes generated from PD-iPSC (i.e. mutant LRRK2 cases) in the absence of neurons exhibit much higher levels of endogenous α-synuclein expression than astrocytes generated from control-iPSC cases [62]. Astrocytes can uptake α-synuclein by endocytosis and accumulate it [40] or degrade it in the lysosome [56]. Our results obtained from iSyn mice (that expresses human A53T-mutant α-synuclein only in neurons) showed no changes in astrocyte Ca^2+^ activity or SICs frequency, indicating that the changes in astrocyte-neuron communication are independent of the up-taken neuronal α-synuclein. Thus, besides taking up α-synuclein from the extracellular media for degradation, our results suggest that astrocytes express α-synuclein that regulates their intrinsic Ca^2+^ signal and gliotransmitter release.

In conclusion, present results show that the A53T point mutation in α-synuclein exacerbates astrocyte Ca^2+^ excitability and astrocyte-neuron signaling independently of neurotransmission or neuronal α-synuclein expression. Therefore, our results indicate that dysfunctions in astrocyte Ca^2+^ excitability and astrocyte-neuron signaling may contribute to the pathophysiology of α-synucleinopathies.

## Supplementary Information

Below is the link to the electronic supplementary material.

Supplementary file1 (PDF 455 KB)

## Data Availability

Any reasonable requests for raw data generated and analyzed for this manuscript are available from the corresponding authors upon reasonable request. TgA53T mouse line (G2-3) and SNCA-KO mice are available from Jackson lab. Other mouse lines are available from Dr. Michael Lee (mklee@umn.edu) with the execution of material transfer agreement from Johns Hopkins University and University of Minnesota.
