# Correction to: Dysregulation of astrocytic Ca^2+^ signaling and gliotransmitter release in mouse models of α-synucleinopathies

**DOI:** 10.1007/s00401-023-02573-1

**Published:** 2023-04-21

**Authors:** Carmen Nanclares, Jonah Poynter, Hector A. Martell-Martinez, Scott Vermilyea, Alfonso Araque, Paulo Kofuji, Michael K. Lee, Ana Covelo

**Affiliations:** 1 Department of Neuroscience, University of Minnesota, 4-125 Jackson Hall, 321 Church Street SE, Minneapolis, MN 55455 USA; 2 Institute for Translational Neuroscience, University of Minnesota, 2101 6th Street SE, Minneapolis, MN 55455 USA; 3 Institut National de la Santé et de la Recherche Médicale (INSERM), U1215 NeuroCentre Magendie, 33077 Bordeaux, France; 4 University of Bordeaux, 33077 Bordeaux, France

**Correction to: Acta Neuropathologica** 10.1007/s00401-023-02547-3

The article Dysregulation of astrocytic Ca^2+^ signaling and gliotransmitter release in mouse models of α‑synucleinopathies, written by Carmen Nanclares, Jonah Poynter, Hector A. Martell‑Martinez, Scott Vermilyea, Alfonso Araque, Paulo Kofuji, Michael K. Lee, Ana Covelo, was originally published electronically on the publisher’s internet portal on 10 February 2023 without open access. With the author(s)’ decision to opt for Open Choice the copyright of the article changed on 3 April 2023 to © The Author(s) 2023 and the article is forthwith distributed under the terms of the “Creative Commons Attribution 4.0 International License, which permits use, sharing, adaptation, distribution and reproduction in any medium or format, as long as you give appropriate credit to the original author(s) and the source, provide a link to the Creative Commons licence, and indicate if changes were made. The images or other third party material in this article are included in the article’s Creative Commons licence, unless indicated otherwise in a credit line to the material. If material is not included in the article’s Creative Commons licence and your intended use is not permitted by statutory regulation or exceeds the permitted use, you will need to obtain permission directly from the copyright holder. To view a copy of this licence, visit http://creativecommons.org/licenses/by/4.0/”.

The original article has been corrected.

