# Supplementary material for: Dysregulation of astrocytic Ca2+ signaling and gliotransmitter release in mouse models of α-synucleinopathies
Source: Acta Neuropathol. 2023 Feb 10;145(5):597–610. doi: 10.1007/s00401-023-02547-3 (PMC10119048; doi:10.1007/s00401-023-02547-3)
Supplement: Supplementary file 1 — Supplementary file1 (PDF 455 KB) [file 401_2023_2547_MOESM1_ESM.pdf]

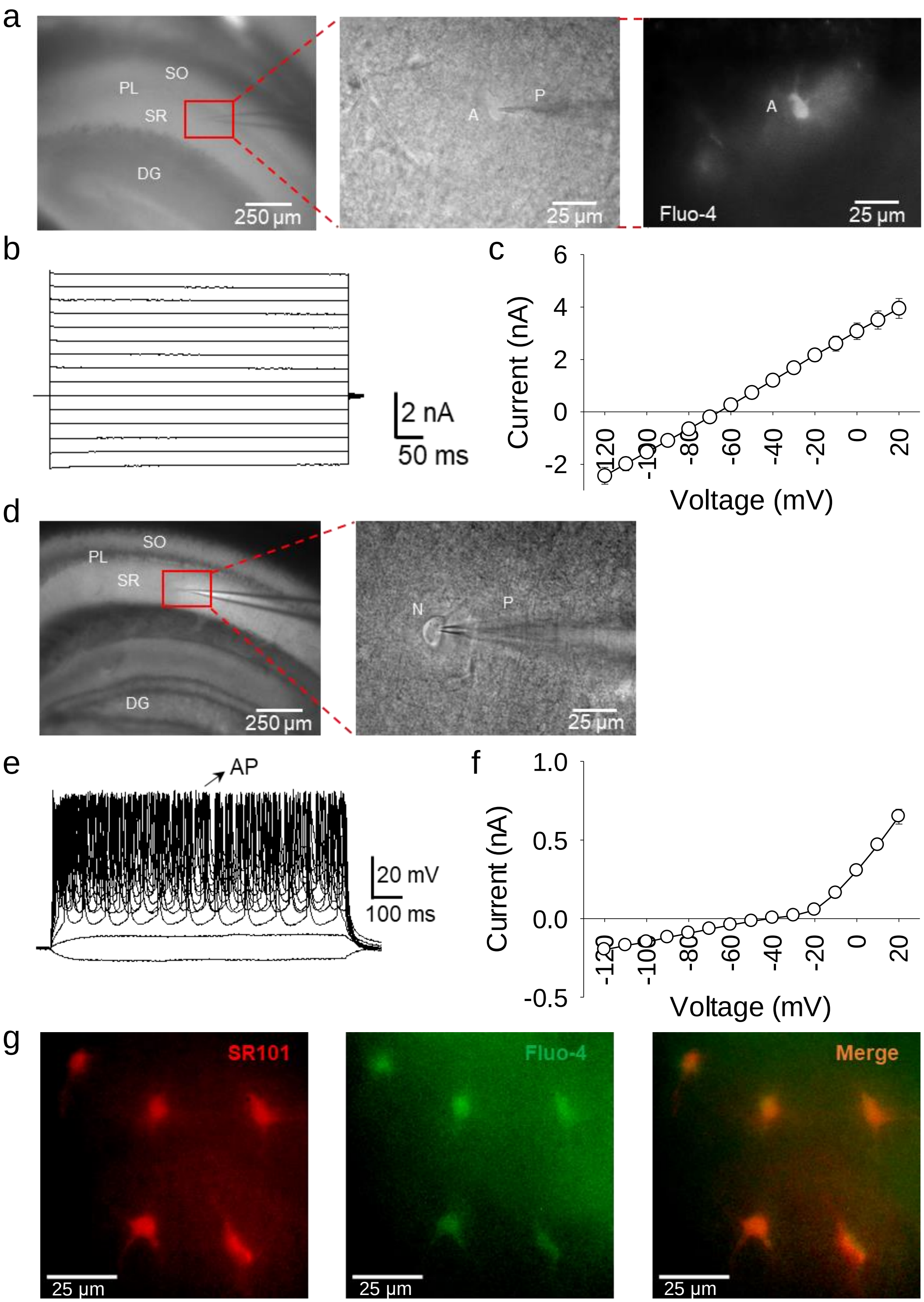

Suppl. Fig. 1 Fluo-4 specifically labels astrocytes. a) Differential interference contrast (DIC) image of a coronal hippocampal section of a WT mouse (left), magnification of the framed region (middle) and Fluo-4 image of the cell patched (right). A: Astrocyte. P: Pipette. b) Representative trace of an intensity vs voltage relationship from an astrocyte recorded in the *stratum radiatum* (Fluo-4 positive). Note the linear relationship and the absence of action potentials. c) Average of intensity vs voltage relationship obtained from 9 Fluo-4 positive cells. d) DIC image of a coronal hippocampal section of a WT mouse (left) and magnification of the framed region (right). N: Interneuron. P: Pipette. e) Representative trace of an intensity vs voltage relationship from an interneuron recorded in the *stratum radiatum* (Fluo-4 negative). Note that the relationship is not linear and the firing of action potentials (AP). f) Average of intensity vs voltage relationship obtained from Fluo-4 negative cells. g) Cells labeled in red with the astrocyte marker SR101 (left), in green with Fluo-4 (middle) and the merge of the two channels (right). SO: *stratum oriens*; PL: *pyramidal layer*; SR: *stratum radiatum*; DG: *dentate gyrus*.

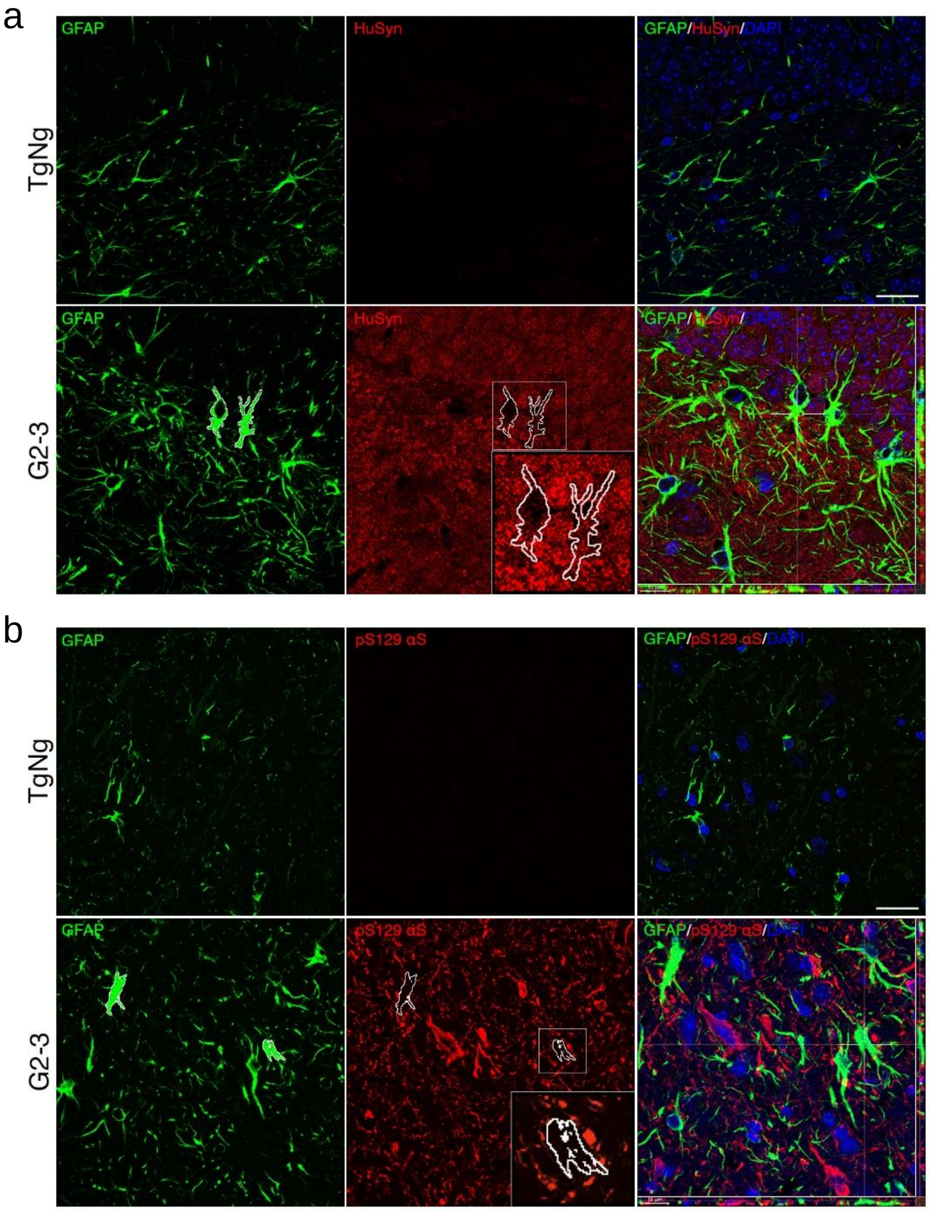

Suppl. Fig. 2 Astrocytes in G2-3 mice express human A53T α-synuclein but do not develop intracellular pS129 α-synuclein aggregates. a) Astrocytes within the *dentate gyrus* of G2-3 mice, but not TgNg mice, contain intracellular human α-synuclein. b) While TgNg mice do not develop pS129 α-synuclein, activated astrocytes residing in the brain stem of G2-3 end stage mice, where pS129 α-synuclein is abundant, do not present observable intracellular pS129 α-synuclein aggregates. Abbreviations: Transgenic negative; TgNg; glial fibrillary acidic protein, GFAP; human alpha-synuclein, HuSyn. Scale bars = 25 μm.
